# Promiscuous Gene Expression in the Thymus: The Root of Central Tolerance

**DOI:** 10.1080/17402520600877091

**Published:** 2006

**Authors:** Danielle A. R. Magalhães, Eduardo L. V. Silveira, Cristina M. Junta, Paula Sandrin-Garcia, Ana Lucia Fachin, Eduardo A. Donadi, Elza T. Sakamoto-Hojo, Geraldo A. S. Passos

**Affiliations:** 1 Molecular Immunogenetics Group Department of Genetics Faculty of Medicine University of São Paulo (USP) Ribeirão Preto SP 14040-900 Brazil; 2 Division of Clinical Immunology Department of Medicine Faculty of Medicine USP Ribeirão Preto SP 14040-900 Brazil; 3 Laboratory of Cytogenetics and Mutagenesis Department of Genetics Faculty of Medicine USP Ribeirão Preto SP 14040-900 Brazil; 4 Department of Biology (FFCLRP) USP Ribeirão Preto SP 14040-900 Brazil; 5 Discipline of Genetics Department of Morphology Faculty of Dentistry USP Ribeirão Preto SP 1404-900 Brazil

## Abstract

The thymus is a complex organ with an epithelium formed by two main cell types, the cortical thymic epithelial (cTECs) and medullary thymic epithelial cells (mTECs), referred to as stroma. Immature thymocytes arising from the bone marrow, macrophages and dendritic cells also populate the thymus. Thymocytes evolve to mature T cells featuring cell differentiation antigens (CDs), which characterize the phenotypically distinct stages, defined as double-negative (DN), double positive (DP) and single positive (SP), based on expression of the coreceptors CD4 and CD8. The thymus is therefore implicated in T cell differentiation and during development into T cells thymocytes are in close association with the stroma. Recent evidence showed that mTECs express a diverse set of genes coding for parenchymal organ specific proteins. This phenomenon has been termed promiscuous gene expression (PGE) and has led to the reconsideration of the role of the thymus in central T cell tolerance to self-antigens, which prevents autoimmunity. The evidence of PGE is causing a reanalysis in the scope of central tolerance understanding. We summarize the evidence of PGE in the thymus, focusing particularly the use of cDNA microarray technology for the broad characterization of gene expression and demarcation of PGE emergence during thymus ontogeny.


*Clinical & Developmental Immunology*, June–December 2006; 13(2–4): 81–99

Taylor & Francis

Taylor & Francis Group

### Promiscuous gene expression in the thymus: The root of central tolerance

DANIELLE A. R. MAGALHÃES[1], EDUARDO L. V. SILVEIRA[1], CRISTINA M. JUNTA[1], PAULA SANDRIN-GARCIA[1], ANA LUCIA FACHIN[1], EDUARDO A. DONADI[2], ELZA T. SAKAMOTO-HOJO[3,4], & GERALDO A. S. PASSOS[1,5]

[1]*Molecular Immunogenetics Group, Department of Genetics, Faculty of Medicine, University of São Paulo (USP), 14040-900 Ribeirão Preto, SP, Brazil,* [2]*Division of Clinical Immunology, Department of Medicine, Faculty of Medicine, USP, 14040-900 Ribeirão Preto, SP, Brazil,* [3]*Laboratory of Cytogenetics and Mutagenesis, Department of Genetics, Faculty of Medicine USP, 14040-900 Ribeirão Preto, SP, Brazil,* [4]*Department of Biology (FFCLRP), USP, 14040-900 Ribeirão Preto, SP, Brazil, and* [5]*Discipline of Genetics, Department of Morphology, Faculty of Dentistry, USP, 14040-900, Ribeirão Preto, SP, Brazil*

**Abstract**
The thymus is a complex organ with an epithelium formed by two main cell types, the cortical thymic epithelial (cTECs) and medullary thymic epithelial cells (mTECs), referred to as stroma. Immature thymocytes arising from the bone marrow, macrophages and dendritic cells also populate the thymus. Thymocytes evolve to mature T cells featuring cell differentiation antigens (CDs), which characterize the phenotypically distinct stages, defined as double-negative (DN), double positive (DP) and single positive (SP), based on expression of the coreceptors CD4 and CD8. The thymus is therefore implicated in T cell differentiation and during development into T cells thymocytes are in close association with the stroma. Recent evidence showed that mTECs express a diverse set of genes coding for parenchymal organ specific proteins. This phenomenon has been termed promiscuous gene expression (PGE) and has led to the reconsideration of the role of the thymus in central T cell tolerance to self-antigens, which prevents autoimmunity. The evidence of PGE is causing a reanalysis in the scope of central tolerance understanding. We summarize the evidence of PGE in the thymus, focusing particularly the use of cDNA microarray technology for the broad characterization of gene expression and demarcation of PGE emergence during thymus ontogeny.

**Keywords:** *Autoimmunity, cDNA microarray, promiscuous gene expression, self–non-self discrimination, T cell tolerance, thymus*

### Considerations on self–non-self discrimination

Self–non-self discrimination is an essential property of the immune system, which contributes to body homeostasis. The germinal idea of clonal selection theory and self-discrimination was developed by Paul Ehrlich more than 100 years ago (Ehrlich and Morgenroth 1901) and represents up to the present, the basic conceptual orientation for all immunological research.

In this regard, the direct demonstration of the clonal deletion of self-reactive lymphocytes is among the most important achievements, which has contributed to our contemporary understanding of self-tolerance in adaptive (Schwartz and Mueller 2003) and in innate (Medzhitov and Janeway 1997) immune systems (for further reading, see review of Kyewski and Derbinski 2004).

The two known pathways of immune response are characterized by the use of limited germline-encoded

Correspondence: G. A. S. Passos, Molecular Immunogenetics Group, Department of Genetics, Faculty of Medicine, University of São Paulo (USP), 14040-900, Ribeirão Preto, SP, Brazil. Tel: 55 16 36 02 30 30. Fax: 55 16 36 33 00 69. E-mail: passos@rge.fmrp.usp.br

ISSN 1740-2522 print/ISSN 1740-2530 online © 2006 Taylor & Francis
DOI: 10.1080/17402520600877091

receptors for microbial components in the innate immune system or by highly diverse, somatically generated (somatic DNA rearrangements) antigen-specific T cell receptor (TCR) or immunoglobulin (Ig) receptors in adaptive immune system.

As an intellectual exercise, the heterodimeric TCR molecule (e.g. $\alpha/\beta$ TCR) could materialize the functional concept of the immune system due to their property in instantly recognizing the self major histocompatibility complex (MHC) and the non-self antigenic peptide.

Self-tolerance of the T cell repertoire is acquired during the development of immature T cells, a phenomenon dependent on the avidity of the interaction between specific TCR and self-peptide-MHC ligands. The nascent T cell repertoire in the thymus determines the diversity of self-antigens and the specificity of central self-tolerance. We agree with Kyewski and Derbinski (2004) in subsuming under central tolerance the intrathymic mechanisms, which T cells undergo in recognition of self-antigens. Accordingly, self-reactive regulatory T ($T_{reg}$) cells are selected in the thymus, even though these cells play their role in the periphery.

In fact, all subsets of antigen-presenting cells (APCs), such as cortical thymic epithelial cells (cTECs), medullary thymic epithelial cells (mTECs), thymic dendritic cells and macrophages play their roles in presenting unique sets of self-peptides, contributing to the diversity of self-antigens displayed in the thymus (Klein and Kyewski 2000).

However, the expression of tissue-specific antigens (TSAs) in the thymus, which represents a key feature for effecting self-representation, was only recently recognized. The evidence of thymic expression of TSAs in mice and humans, which has been referred to as promiscuous gene expression (PGE) (Jolicoeur et al. 1994; Derbinski et al. 2001; Gotter et al. 2004), reinforced the conception of central tolerance of tissue-specific self-antigens.

Prior to this evidence, the peripheral tolerance, that is, the mechanisms by which T cell selection towards TSAs occurs outside the thymus, dominated the scenario in explaining self–non-self discrimination (Alferink et al. 1999; Walker and Abbas 2002).

Promiscuous gene expression in the thymus is causing a reversal in the scope of central tolerance understanding, allowing for an unorthodox conception of the possible mechanisms of self–non-self discrimination (Kyewski et al. 2002; Gallegos and Bevan 2006).

The main implication of this heterogeneous gene expression in the thymus is associated with the maintenance of the immunological homeostasis in the body, controlling the pathogenic autoimmune reactions.

Evidence for this phenomenon was obtained using the reverse transcription-PCR (RT-PCR) method, which was biased towards antigens in autoimmune reactions, such as insulin, acetylcholine receptor or myelin basic protein. Today it is recognized that rather than selective, the set of expressed genes is as broad as possible, estimated to include up to 5–10% of all currently known mouse genes (Derbinski et al. 2001; Gotter et al. 2004; Kyewski and Derbinski 2004).

### Using the microarray technology

At present, to explore the broad gene expression in the thymus, the choice strategy is the use of the microarray technology. The group of Kyewski and Derbinski (2004) from the German Cancer Research Centre in Heidelberg, Germany, has used the Affymetrics oligonucleotide microarray platform, which has allowed for extensive characterization of PGE in the mouse thymus.

In fact, large scale gene expression measurements by microarrays, in their different formats, such as, nylon membranes or glass slides have been used for more than 10 years to study the control of gene expression in the thymus, focusing mainly on thymocytes (Nguyen et al. 1995; Espanhol et al. 2004; Puthier et al. 2004; Magalhães et al. 2005; Cardoso et al. 2006).

The control of gene expression during the development of this organ has gained priority among several research teams, including our own group, allowing for the identification of candidate genes involved in thymopoiesis (Espanhol et al. 2004; Magalhães et al. 2005; Cardoso et al. 2006). A number of expressed sequence tags (ESTs) have been found that are modulated during the *in vivo* development of the thymus (Espanhol et al. 2004) and several cell-signaling genes, including those of the calcium cascade pathway, which is important for individual stages of T cell maturation and the control of anergy during thymus ontogeny (Magalhães et al. 2005).

Regarding in-lab cDNA microarray preparation for PGE research purposes in the mouse, of particular interest is the Soares thymus 2NbMT cDNA library constructed by Maria F. Bonaldo and Marcelo B. Soares of the Columbia University, New York, USA, in cooperation with Bertrand Jordan of the Centre d'Immunologie de Marseille-Luminy, France, whose researchers have made this library available at the IMAGE Consortium (http://image.llnl.gov). This is an EST normalized library, prepared from a C57Bl/6J 4 week-old male thymus and whose cDNA inserts, ranging from 0.5 to 1.5 kb in length, were cloned in the pT7T3D vector.

The library is composed of more than 25,000 resequenced clones representing most, if not the whole set of expressed genes by the mouse thymus, including those representing parenchymal and lymphoid organs. Thus, it represents a precious resource for preparing

"specialized" thymus cDNA microarrays for further use in promiscuous gene expression determinations.

The work on gene expression of fetal mouse thymus realized by our group is performed using two cDNA microarray platforms; the first version is prepared on a glass slide containing 4500 target sequences per slide, which are hybridized with fluorescent cDNA probes labeled with Cy3 or Cy5. The second version is prepared on positively charged nylon membranes containing 750 target sequences, which are hybridized with radioactive cDNA probes labeled with $^{33}P$ isotope.

We defined as "complex probe", the labeled cDNA originated from thymus total RNA and as "target", the cloned sequences deposited in individual spots on the microarrays.

All target cDNA clones deposited on these two versions of microarrays are from the thymus 2NbMT normalized library mentioned above, which were previously amplified by PCR in 384- or 96-well plates using vector-PCR amplification with the following primers, which recognize the cloning vector: LBP 1S GTGGAATTGTGAGCGGATACC forward and LBP 1AS GCAAGGCGATTAAG-TTGG reverse.

For both versions of microarrays, a Generation III array spotter (Amersham—Molecular Dynamics—Sunnyvale, CA, USA) is used according to the manufacturer's instructions and cross-linked using an ultraviolet cross-linker.

The cDNA probes are prepared by reverse transcription using 10 μg of total RNA from fetal or adult thymus, which are labeled with Cy3 or Cy5 fluorochromes using the CyScribe post labeling kit (GE Healthcare, USA) and oligonucleotide $dT_{12-18}$ as a primer. The 15 h period required for glass slide hybridization followed by washing is performed at 42°C in an automated slide processor (ASP, Amersham Biosciences) and the microarrays are scanned in a Generation III laser scanner (Amersham Biosciences).

The cDNA complex probes derived from fetal or adult thymus are Cy5-labeled. A Cy3-labeled cDNA pool, originated from a mix of equimolar amount of total RNA from different fetal organs (brain, liver, intestines and spleen), is used as reference in the two-color hybridizations.

The nylon cDNA microarrays are hybridized with radiolabeled $^{33}P$-cDNA complex probes in a rolling oven at 65°C for 72 h and scanned in a phosphor imager storage system (Cyclone model, Packard Instruments, USA).

The characterization of each cDNA sequence is updated using the SOURCE genome data bank (http://genome-www5.stanford.edu/cgi-bin/source/sourceSearch), providing information such as DNA and protein sequences, biological and molecular functions and chromosomal location.

### Preparing thymus tissue and total RNA for PGE experiments

The classic Balb-c, C57Bl/6 and (♀Balb-c × ♂C57Bl/6)$F_1$ mouse strains should be preferentially bred in an isolator with 0.45 μm pore sized air filtered. To obtain timed pregnancies of Balb-c, C57Bl/6 and their hybrids at the developmental phase when TRBV8.1 V(D)J recombination occurs, the mice are mated and the day when the vaginal plug is observed at 7:00 am is considered to be day zero of gestation post coitum (p.c.). The pregnant mice are sacrificed preferentially by $CO_2$ inhalation and the fetuses collected by surgery of the uterus. The p.c. age of fetuses should be confirmed by observing the morphological characteristics of each developmental phase (Rugh 1968).

The onset of TCR beta V(D)J recombination (TRBV8.1-BD2.1) during the *in vivo* fetal development of the thymus of Balb-c, C57Bl/6 and their hybrids is taken on the basis of previously published data. Fetal thymus should be obtained at the emergence of V(D)J recombination, that is, at 15–16 days p.c. for Balb-c and 14–15 days p.c. for C57Bl/6 and (♀Balb-c × ♂C57Bl/6)$F_1$ (Macedo et al. 1999; Magalhães et al. 2005).

The thymi should be removed from the fetuses preferable under a stereomicroscope and the epithelial cells preparation enriched on Percoll® gradient centrifugation or purified by cell sorting, which are immediately processed for total RNA extraction.

In the microarray experiments, it is extremely important to use only undegraded and DNA-, protein- and phenol-free RNA preparations as verified by classic agarose gel electrophoresis stained with ethidium bromide and ultraviolet spectrophotometry, respectively.

### Analysing microarray data by statistical significance algorithm

The gene expression data obtained from microarray experiments should be analysed by a mathematical/-statistical algorithm. In PGE projects, we are using the significance analysis of microarrays method (SAM, available online at http://www-stat.stanford.edu/~tibs/SAM/index.html) to analyse the significant variations in gene expression (Tusher et al. 2001). This method is based on *t*-test statistics, specially modified for high throughput analysis. We propose discussing only those genes which present a fold change expression ≥ 1 (induced) and a false discovery rate (FDR) max. 0.05 (Table I, available online at http://rge.fmrp.usp.br/passos/review/PGE/table1).

To analyse the significant variations in the gene expression of developing thymus, RNA samples are extracted on fixed days p.c. are compared with RNA from subsequent days of gestation.

Table I. Genes differentially and significantly induced in the thymus of C57Bl/6 and (♀Balb-c × ♂C57Bl/6)$F_1$ as detected by the SAM program. Fold change ≥ 1 and FDR, false discovery rate. Magalhães et al. (2006) promiscuous gene expression in the thymus.

| Gene name | GenBank accession | Chromosomes | Predominant expression | Molecular/biological function |
|---|---|---|---|---|
| *C57Bl/6 strain (fold change = 1.5 and FDR ~ 0.05)* | | | | |
| Hexosaminidase B (Hexb) | NM_010422 | 13 | Adipose tissue, bone, amygdale, frontal cortex, trigeminal, cerebral cortex, dorsal root ganglia, dorsal striat um, hippocampus, hypothalamus, olfactory bulb, spinal cord'lower, spinal cord upper, substantia nigra, blatocystis, placenta, small intestine, B220+B-cells, lymphnode, vomeronasal organ, salivary gland, pituitary, snout epidermis, thymus, thyroid, trachea, bladder, kidney | Metabolism |
| Bromodomain containing 4 (Brd4) | NM_020508 | 17 | Adipose tissue, bone, bonemarrow, amygdala, preoptic, hippocampus, spinal cord'lower, embryo day 10.5, embryo day 6.5, embryo day 7.5, embryo day 8.5, embryo day 9.5, ovary, prostate, umbilical cord, uterus, heart, small intestine, B220+B-cells, CD4+ Tcells, CD8+ T cells, lung, lymphnode, pancreas, digits, snout epidermis, thymus, trachea | Required maternally for proper expression of other homeotic genes involved in pattern formation, such as ubx |
| Stimulated by retinoic acid 13 (Stra13) | NM_016665 | 11E2 | Adipose tissue, adrenalgland, bone, main olfactory epithelium, embryo day 6.5, embryo day 9.5, fertilized egg, ovary, prostate, testis, umbilical cord, uterus, oocyte, large intestine, B220+B-cells, CD4+ T cells, CD8+ T cells, liver, lung, vomeronasal organ, salivary gland, tongue, pituitary, digits, snout epidermis, spleen, thymus, trachea, kidney | – |
| *(♀Balb-c × ♂C57Bl/6)$F_1$ (fold change = 1.0 and FDR ~ 0.0012)* | | | | |
| Ets2 repressor factor (Erf) | NM_010155 | 7 | Brown fat, bonemarrow, dorsal striatum, hippocampus, blastocysts, embryo day 10.5, embryo day 6.5, embryo day 7.5, embryo day 8.5, embryo day 9.5, fertilized egg, mammary gland (lact), ovary, placenta, heart, small intestine, liver, skeletal muscle, salivary gland, pancreas, spleen, stomach, thyroid, bladder | Transcriptional repressor (by similarity) |
| RAB2, member RAS oncogene family (Rab2) | NM_021518 | 4A1 | Adrenal gland, amygdala, frontal cortex, preoptic, trigeminal, cerebellum, cerebral cortex, dorsal root ganglia, dorsal striatum, hippocampus, hypothalamus, main olfactory ephitelium, olfactory bulb, spinal cord lower, spinal cord upper, substantia nigra, fertilized egg, ovary, prostate, oocyte, large intestine, lung, skeletal muscle, vomeronasal organ, pituitary, digits, epidermis, bladder, kidney, retina | Required for protein transport from the endoplasmic reticulum to the golgi complex (by similarity) |

Table I – *Continued*

| Gene name | GenBank accession | Chromosomes | Predominant expression | Molecular/biological function |
|---|---|---|---|---|
| Membrane associated DNA binding protein (Mnab) | XM_130233 | 2 | Adrenal gland, amygdala, frontal cortex, preoptic, trigeminal, cerebellum, cerebral cortex, dorsal root ganglia, dorsal striatum, hippocampus, hypothalamus, main olfactory ephitelium, olfactory bulb, spinal cord lower, substantia nigra, embryo day 10.5, ovary, prostate, umbilical cord, lung, skeletal muscle, vomeronasal organ, tongue, pituitary, digits, epidermis, thymus, trachea, bladder, retina | Nucleic acid binding |
| RAD51-like 1 (S. cerevisiae) (Rad51l1) | NM_009014 | 12 C3 | Brown fat, bonemarrow, dorsal striatum, embryo day 9.5, fertilized egg, mammary gland (lact), ovary, placenta, testis, uterus, B220+B-cells, CD4+ Tcells, salivary gland, pancreas, spleen, stomach, thyroid, trachea | DNA repair (by similarity) |
| Small chemokine (C–C motif) ligand 11 (Ccl11) | NM_011330 | 11 | Brown fat, adipose tissue, trigeminal, ovary, prostate, uterus, large intestine, small intestine, lymphnode, skeletal muscle, tongue, digits, epidermis, snout epidermis, stomach, thymus, thyroid, trachea, bladder | Immune response by eosinophils |
| DEAD (Asp-Glu-Ala-Asp) box polypeptide 21 (Ddx21) | NM_019553 | 10 | Adipose tissue, adrenal gland, bone, blastocysts, embryo day 10.5, embryo day 6.5, embryo day 7.5, embryo day 8.5, embryo day 9.5, mammary gland (lact), ovary, prostate, umbilical cord, uterus, larger intestine, B220+B-cells, CD4+ Tcells, CD8+ Tcells, lung, lymphnode, salivary gland, tongue, digits, epidermis, spleen, thymus, trachea, bladder | Nucleic acid binding RNA helicase/foldase (by similarity) |
| Sodium channel modifier 1 (Scnm1) | NM_027013 | 3 | Brown fat, frontal cortex, preoptic, trigeminal, hippocampus, hypothalamus, main olfactory ephitelium, spinal cord lower, substantia nigra, blastocysts, embryo day 10.5, embryo day 6.5, embryo day 7.5, embryo day 9.5, ovary, testis, heart, B220+B-cells, CD4+ Tcells, CD8+ Tcells, tongue, pituitary, digits, snout epidermis, thymus, trachea | Ion channel activity and RNA splicing |
| Sialophorin (Spn) | NM_009259 | 7 | Bone, bonemarrow, embryo day 10.5, embryo day 6.5, embryo day 7.5, embryo day 9.5, placenta, B220 + B-cells, CD4+ Tcells, CD8+ Tcells, lung, lymphnode, spleen, thymus, trachea | Negative regulatory role in adaptive immune response (by similarity) |
| Adenosine deaminase (Ada) | NM_007398 | 2 | Placenta, small intestine, tongue, thymus, trachea | Immune response, nucleotide metabolism |
| Ecotropic viral integration site 2[a] (Evi2a) | NM_010161 | 11 | Brown fat,bone, bonemarrow, dorsal striatum, hippocampus, spinal cord upper, blastocysts, embryo day 10.5, embryo day 6.5, embryo day 7.5, embryo day 9.5, mammary gland (lact), oocyte, heart, CD8+ Tcells, liver, lymphnode, pancreas, epidermis, spleen, stomach, thymus, thyroid, trachea | – |

Table I – *Continued*

| Gene name | GenBank accession | Chromosomes | Predominant expression | Molecular/biological function |
|---|---|---|---|---|
| B-cell leukemia/lymphoma 6 (Bcl6) | NM_009744 | 16 | Adipose tissue, adrenal gland, bone, cerebellum, hippocampus, main olfactory ephitelium, olfactory bulb, spinal cord lower, spinal cord upper, ovary, prostate, uterus, B220+B-cells, lymphnode, skeletal muscle, salivary gland, digits, epidermis, snout epidermis, thymus, trachea, kidney, retina | Transcription factor |
| Interleukin 16 (Il16) | NM_010551 | 7 | Adipose tissue, cerebellum, B220+B-cells, CD4+ Tcells, CD8+ Tcells, lymphnode, spleen, thymus, trachea | Cytokine activity, immune cell chemotaxis |
| Chemokine (C–X–C motif) ligand 4 (Cxcl4) | NM_019932 | 5 E1 | Adipose tissue, bone, bonemarrow, trigeminal, ovary, umbilical cord, heart, lung, skeletal muscle, vomeronasal organ, tongue, digits, epidermis, snout epidermis, spleen, trachea, bladder | Platelet factor 4, is released during platelet aggregation |
| Microtubule-associated protein, RP/EB family, member 2 (Mapre2) | NM_153058 | 18 A2 | Brown fat, bonemarrow, dorsal striatum, blastocysts, embryo day 10.5, embryo day 6.5, embryo day 7.5, embryo day 9.5, mammary gland (lact), placenta, prostate, heart, small intestine, liver, lung, lymphnode, skeletal muscle, salivary gland, pancreas, spleen, stomach, thyroid, bladder | Microtubule binding and protein binding |
| WAP, follistatin/kazal, immunoglobulin, kunitz and netrin domain containing 2 (Wfikkn2) | NM_181819 | 11D | Adipose tissue, prostate, umbilical cord, uterus, CD4+ Tcells, CD8+ Tcellslung, lymphnode, longue, digits, epidermis, thymus | Alpha-1-microglobulin occurs in many physiological fluids including plasma, urine, and cerebrospinal fluid (by similarity) |
| Signal transducer and activator of transcription 1 (Stat1) | NM_009283 | 1 | Adipose tissue, adrenal gland, bone, bonemarrow, trigeminal, dorsal root ganglia, ovary, uterus, B220+B-cells, CD4+ Tcells, CD8+ Tcells, lung, lymphnode, pancreas, spleen, thymus, thyroid, trachea | Transcription factor |
| C-type lectin domain family 1, member b (Clec1b) (Clec2) | NM_019985 | 6F3 | Bone, bonemarrow, embryo day 10.5, placenta, liver, lymphnode, pancreas, spleen, trachea | Cell surface receptor linked signal transduction |
| Exportin, tRNA (nuclear export receptor for tRNAs) (Xpot) | XM_125902 | 10D3 | Adrenal gland, amygdale, frontal cortex, preoptic, cerebellum, cerebral cortex, dorsal root ganglia, hippocampus, hypothalamus, olfactory bulb, spinal cord lower, spinal cord upper, substantia nigra, blastocysts, embryo day 10.5, embryo day 6.5, embryo day 7.5, embryo day 9.5, mammary gland (lact), ovary, prostate, umbilical cord, uterus, lung, longue, digits, thymus, trachea | Mediates nuclear export of all tRNAs (by similarity) |
| Ubiquitin-activating enzyme E1, Chr X (Ube1x) | NM_009457 | X | Adrenal gland, preoptic, trigeminal, cerebellum, cerebral cortex, dorsal root ganglia, olfactory bulb, substantia nigra, blastocysts, embryo day 10.5, embryo day 6.5, embryo day 9.5, fertilized egg, ovary, prostate, uterus, oocyte, CD4+ Tcells, longue, digits, snout epidermis, thymus, trachea | ATP binding |

Table I – *Continued*

| Gene name | GenBank accession | Chromosomes | Predominant expression | Molecular/biological function |
|---|---|---|---|---|
| Activating transcription factor 4 (Atf4) | NM_009716 | 15 | Adrenal gland, cerebellum, main olfactory epithelium, blastocysts, embryo day 6.5, embryo day 7.5, embryo day 9.5, placenta, umbilical cord, uterus, oocyte, large intestine, B220+B-cells, CD4+ Tcells, lung, skeletal muscle, vomeronasal organ, salivary gland, tongue, pituitary, digits, snout epidermis, thymus, trachea, retina | Transcription factor |
| Zinc finger protein 131 (Zfp131) | NM_028245 | 13 | Adipose tissue, adrenal gland, bone, bonemarrow, amygdale, frontal cortex, cerebellum, embryo day 9.5, fertilized egg, ovary, uterus, oocyte, large intestine, B220+B-cells, CD4+ Tcells, CD8+ Tcells, lung, lymphnode, skeletal muscle, digits, thymus, trachea | May be involved in transcriptional regulation |
| RAB14, member RAS oncogene (Rab14) | NM_026697 | 2B | Adipose tissue, bone, bonemarrow, preoptic, blastocysts, ovary, umblical cord, lymphnode, longue, digits, epidermis, snout epidermis, thymus*, trachea | Required for protein transport in the secretory pathway |
| Synaptophysin-like protein (Sypl) | NM_198710 | 12 | Adipose tissue, adrenalgland, bone, main olfactory epithelium, spinal cord'lower, spinal cord upper, fertilized egg, ovary, placenta, prostate, uterus, oocyte, heart, large intestine, small intestine, B220+ B cells, lung, skeletal muscle, vomeronasal organ, salivary gland, tongue, digits, epidermis, snout epidermis, stomach, trachea, bladder, kidney, retina | Transport, transporter activity |
| WD repeat and SOCS box-containing 2 (Wsb2) | NM_021539 | 5F | Adrenal gland, amygdale, frontal cortex, preoptic, trigeminal, cerebellum, cerebral cortex, dorsal root ganglia, dorsal striatum, hippocampus, hypothalamus, main olfactory ephitelium, olfactory bulb, spinal cord lower, spinal cord upper, substantia nigra, ovary, placenta, umbilical cord, uterus, large intestine, lymphnode, skeletal muscle, vomeronasal organ, pancreas, pituitary, spleen, kidney | Intracellular signaling cascade |
| POU domain, class 2, associating factor 1 (Pou2af1) | NM_011136 | 9 A5.3 | Adipose tissue, bone, bonemarrow, B220+B-cells, CD4+ Tcells, CD8+ Tcells, lymphnode, spleen, trachea | DNA binding, regulation of transcription |
| Transformed mouse 3T3 cell double minute 2 (Mdm2) | NM_010786 | 10 | Adrenal gland, amygdale, preoptic, spinal cord lower, substantia nigra, blastocysts, embryo day 6.5, embryo day 8.5, embryo day 9.5, fertilized egg, placenta, testis, oocyte, B220+B-cells, CD4+ Tcells, CD8+ Tcells, lung, lymphnode, skeletal muscle, vomeronasal organ, thymus, trachea | Cell growth and/or maintenance |
| Interleukin 4 (Il4) | NM_021283 | 11 | Brown fat, bonemarrow, dorsal striatum, hippocampus, embryo day 10.5, embryo day 7.5, embryo day 9.5, fertilized egg, mammary gland (lact), placenta, testis, heart, large intestine, small intestine, liver, skeletal muscle, salivary gland, longue, pancreas, spleen, stomach, thyroid, bladder, kidney | Participates in at least several b-cell activation processes as well as of other cell types |

Table I – *Continued*

| Gene name | GenBank accession | Chromosomes | Predominant expression | Molecular/biological function |
|---|---|---|---|---|
| Protein O-fucosyltransferase 2 (Pofut2) | NM_030262 | 10C1 | Brown fat, Adipose tissue, adrenal gland, preoptic, cerebellum, main olfactory epithelium, embryo day 6.5, embryo day 7.5, embryo day 8.5, embryo day 9.5, fertilized egg, ovary, placenta, testis, umbilical cord, uterus, oocyte, lung, vomeronasal organ, pituitary, snout epidermis | Carbohydrate metabolism |
| CD24a antigen (Cd24a) | NM_009846 | 10 | Adipose tissue, bone, bonemarrow, trigeminal, dorsal root ganglia, main olfactory epithelium, embryo day 10.5, embryo day 8.5, embryo day 9.5, mammary gland (lact), ovary, prostate, uterus, large intestine, B220+B-cells, lung, skeletal muscle, vomeronasal organ, salivary gland, longue, digits, spleen, stomach, thymus, thyroid, trachea, kidney, retina | May have a pivotal role in cell differentiation |
| Transcription factor E2a (Tcfe2a) | NM_011548 | 10 | Adipose tissue, adrenal gland, bone, bonemarrow, cerebellum, main olfactory epithelium, embryo day 10.5, embryo day 6.5, embryo day 7.5, embryo day 8.5, embryo day 9.5, fertilized egg, ovary, umbilical cord, uterus, oocyte, B220+B-cells, CD4+ Tcells, CD8+ Tcells, lymphnode, pituitary, digits, epidermis, snout epidermis, spleen, thymus, trachea, bladder | Transcription factor |
| Interferon-induced protein with tetratrico-peptide repeats 2 (Ifit2) | NM_008332 | 19C1 | Adipose tissue, adrenal gland, bone, retina, bonemarrow, trigeminal, dorsal root ganglia, hypothalamus, main olfactory epithelium, olfactory bulb, spinal cord lower, substantia nigra, ovary, placenta, prostate, uterus, heart, large intestine, B220+B-cells, CD4+ Tcells, CD8+ Tcells, lung, lymphnode, vomeronasal organ, epidermis, spleen, thymus, trachea, kidney | Immune response |
| Cut-like 1 (Drosophila) (Cutil) | NM_009986 | 5 | Brown fat, adrenalgland, bonemarrow, frontal cortex, preoptic, cerebellum, dorsal striatum, main olfactory epithelium, olfactory bulb, spinal cord'lower, substantia nigra, embryo day 10.5, embryo day 7.5, embryo day 8.5, embryo day 9.5, fertilized egg, mammary gland (lact), placenta, umbilical cord, uterus, heart, large intestine, small intestine, liver, lung, skeletal muscle, omeronasal organ, salivary gland, tongue, pancreas, digits, epidermis, snout epidermis, spleen, stomach, thymus, thyroid, bladder, kidney, retina | Probably has a broad role in mammalian development as a repressor of developmentally regulated gene expression |
| Histocompatibility 2, complement com-ponent factor B (H2-Bf) | NM_008198 | 17 | Adipose tissue, ovary, uterus, large intestine, small intestine, liver, lymphnode, digits, epidermis, snout epidermis, spleen, trachea, kidney | Factor B which is part of the alternate pathway of the complement system |
| Fanconi anemia, complementation group G (Fancg) | NM_053081 | 4B1 | Bone, bonemarrow, fertilized egg, testis, oocyte, B220+B-cells, CD4+ Tcells, CD8+ Tcells, salivary gland, longue, digits, snout epidermis, thymus, trachea | Binding,DNA repair, response to DNA damage stimulus, response to radiation, spermatid development |

Table I – *Continued*

| Gene name | GenBank accession | Chromosomes | Predominant expression | Molecular/biological function |
|---|---|---|---|---|
| Ras homolog gene family, member A (Rhoa) | NM_016802 | 9 | Adipose tissue, adrenal gland, bone, dorsal root ganglia, main olfactory epithelium, embryo day 10.5, embryo day 6.5, embryo day 7.5, embryo day 8.5, embryo day 9.5, ovary, prostate, umbilical cord, uterus, heart, large intestine, small intestine, B220+B-cells, CD4+ Tcells, CD8+ Tcells, lung, vomeronasal organ, digits, snout epidermis, stomach, thymus, trachea, bladder, kidney | Regulates a signal transduction pathway linking plasma membrane receptors to the assembly of focal adhesions and actin stress fibers |
| Interferon-induced protein with tetratrico-peptide repeats 1 (Ifit1) | NM_008331 | 19C1 | Brown fat, Adipose tissue, adrenal gland, bone, bone-marrow, trigeminal, dorsal root ganglia, main olfactory epithelium, ovary, placenta, prostate, uterus, heart, large intestine, small intestine, CD4+ Tcells, CD8+ Tcells, liver, lung, lymphnode, vomeronasal organ, longue, pancreas, epidermis, spleen, thymus, trachea, bladder, retina | Immune response |
| Growth factor receptor bound protein 2 (Grb2) | NM_008163 | 11 | Bone, bonemarrow, preoptic, cerebellum, cerebral cortex, dorsal root ganglia, dorsal striatum, hippocampus, hypothalamus, olfactory bulb, spinal cord'lower, sub-stantia nigra, embryo day 10.5, embryo day 6.5, embryo day 7.5, embryo day 8.5, embryo day 9.5, heart, B220+ B cells, CD4+ T cells, CD8+ T cells, lymphnode, tongue, epidermis, snout epidermis | MAPKKK cascade, protein binding, Ras protein signal transduction, SH3/SH2 adaptor activity |
| Histamine receptor H 3 (Hrh3) | NM_133849 | 2H4 | Bone, amygdale, frontal cortex, preoptic, trigeminal, cerebellum, córtex cerebral, dorsal striatum, hippo-campus, hypothalamus, olfactory bulb, spinal cord lower, spinal cord upper, substantia nigra embryo day 7.5, embryo day 9.5, placenta, lung, salivary gland, pancreas, pituitary, spleen, stomach, thyroid, retina | The H3 subclass of histamine receptors could mediate the histamine signals in CNS and peripheral nervous system (by similarity) |
| Tumor necrosis factor receptor superfamily, member 4 (Tnfrsf4) | NM_009452 | 1 | Adipose tissue, main olfactory epithelium, testis, heart, small intestine, B220+B-cells, CD4+ Tcells, lymph-node, skeletal muscle, epidermis, snout epidermis, trachea, kidney | Cellular defense response,inflammatory response |
| Wiskott-Aldrich syndrome protein interact-ing protein (Waspip) | NM_153138 | 2 | Adipose tissue, bone, bonemarrow, dorsal root ganglia, spinal cord lower, blastocysts, embryo day 10.5, mammary gland (lact), umbilical cord, uterus, heart, B220+B-cells, CD4+ Tcells, CD8+ Tcells, lung, lymphnode, vomeronasal organ, longue, digits, snout epidermis, spleen, thymus, trachea, bladder | Actin binding, actin filament-based movement |

Table I – *Continued*

| Gene name | GenBank accession | Chromosomes | Predominant expression | Molecular/biological function |
|---|---|---|---|---|
| BCL2/adenovirus E1B 19 kDa-interacting protein 1, NIP3 (Bnip3) | NM_009760 | 7F5 | Brown fat, Adipose tissue, adrenal gland, frontal cortex, trigeminal, cerebral cortex, dorsal root ganglia, hypo-thalamus, spinal cord lower, spinal cord upper, substantia nigra, blastocysts, embryo day 6.5, embryo day 8.5, ferlized egg, ovary, placenta, prostate, umbilical cord, oocyte, heart, liver, skeletal muscle, vomeronasal organ, longue, pituitary, epidermis, trachea, kidney | Binds to the adenovirus e1b 19 kda protein or to bcl-2. may play a role in repartitioning calcium between the two major intracellular calcium stores in association with the 19 kda or bcl-2 proteins |
| PTK2 protein tyrosine kinase 2 beta (Ptk2b) | NM_172498 | 14 | Adipose tissue, bone, bonemarrow, amygdale, frontal cortex, cerebral cortex, dorsal striatum, hippocampus, olfactory bulb, large intestine, B220+B-cells, CD4+ Tcells, CD8+ Tcells, lymphnode, spleen, thymus, trachea | Involved in calcium induced regulation of ion channel and activation of the map kinase signaling pathway |
| SMT3 suppressor of mif two 3 homolog 3 (yeast) (Sumo3) | NM_019929 | 10 | Bone, amygdale, frontal cortex, preoptic, cerebellum, hypothalamus, main olfactory epithelium, olfactory bulb, spinal cord lower, substantia nigra, , blastocysts, embryo day 6.5, embryo day 8.5, embryo day 9.5, embryo day 10.5 ferlized egg, ovary, prostate, umbilical cord, uterus, oocyte, vomeronasal organ, pituitary, digits, snout epidermis, thymus, trachea, retina | Protein modification, ubiquitin cycle |
| Tumor necrosis factor receptor superfamily, member 13b (Tnfrsf13b) | NM_021349 | 11B2 | Adipose tissue, bone, bonemarrow, B220+B-cells, CD4+ Tcells, CD8+ Tcells, lung, lymphnode, spleen, thymus, trachea | B cell homeostasis, immune response, negative regulation of B cell proliferation |
| Non-catalytic region of tyrosine kinase adaptor protein 2 (Nck2) | NM_010879 | 1C1 | Adipose tissue, adrenal gland, cerebral cortex, hippo-campus, main olfactory epithelium, olfactory bulb, blastocysts, embryo day 9.5, embryo day 10.5, ovary, placenta, prostate, umbilical cord, uterus, heart, large intestine, small intestine, CD4+ Tcells, CD8+ Tcells, lung, vomeronasal organ, tongue, snout epidermis, stomach, thymus, trachea, bladder | Actin filament organization, cell migration, epidermal growth factor receptor signaling pathway |
| T-cell receptor gamma, variable 4 (Tcrg-V4) | Z12299.1 | 13 | Brown fat, Adipose tissue, bonemarrow, dorsal striatum, heart, large intestine, small intestine, CD4+ Tcells, CD8+ Tcells, liver, lymphnode, epidermis, spleen, thymus, trachea | Cellular defense response |
| F-box protein 45 (Fbxo45) | NM_173439 | 16B2 | Adipose tissue, adrenal gland, bone, amygdala, frontal cortex trigeminal, cerebral cortex, cerebellum, dorsal root ganglia, hippocampus, main olfactory epithelium, olfactory bulb, spinal cord lower, substantia nigra, blastocysts, embryo day 9.5, embryo day 10.5, ovary, heart, skeletal muscle vomeronasal organ, tongue, pituitary, digits, epidermis, snout epidermis spleen, thymus, trachea, retina | Ubiquitin cycle |

Table I – *Continued*

| Gene name | GenBank accession | Chromosomes | Predominant expression | Molecular/biological function |
|---|---|---|---|---|
| Protein kinase, interferon inducible double stranded RNA dependent activator (Prkra) | NM_011871 | 2C3 | Adipose tissue, adrenal gland, bone, preoptic, trigeminal, dorsal root ganglia, hypotalamus, spinal cord upper, spinal cord lower, substantia nigra, embryo day 7.5, embryo day 8.5, embryo day 9.5, embryo day 10.5, ovary, testis, umbilical cord, uterus, heart, large intestine, small intestine, liver, lung, pituitary, stomach, trachea, bladder, kidney, retina | Double-stranded RNA binding, kinase activity, protein amino acid phosphorylation |
| Genetic suppressor element 1 (Gse1) | NM_198671 | 8E1 | Bone, bonemarrow, amygdale, preoptic, cerebellum, cerebral cortex, dorsal striatum, hippocampus, hypotalamus, main olfactory epithelium, olfactory bulb, spinal cord lower, substantia nigra, blastocysts, embryo day 10.5, embryo day embryo day 9.5, prostate, oocyte, large intestine, small intestine, B220+B-cells, CD4+ Tcells, lung, lymphnode, pituitary, digits, snout epidermis, thymus, bladder, retina | – |
| Janus kinase 1 (Jak1) | NM_146145 | 4 | Brown fat, Adipose tissue, bone, amygdale, frontal cortex, trigeminal, cerebellum, dorsal striatum, hypotalamus, spinal cord lower, embryo day 7.5, fertilized egg, mammary gland, placenta, uterus, oocyte, heart, B220+B-cells, liver, lymphnode, salivary gland, pancreas, digits, epidermis, spleen, thymus, thyroid | Tyrosine kinase of the non-receptor type, involved in the ifn-alpha/beta/gamma signal pathway |
| Transportin 2 (importin 3, karyopherin beta 2b) (Tnpo2) | NM_145390 | 8C2 | Amygdale, frontal cortex, preoptic, trigeminal, cerebellum, cerebral cortex, dorsal root ganglia, dorsal striatum, hippocampus, hypotalamus, main olfactory epithelium, olfactory bulb, spinal cord upper, spinal cord lower, substantia nigra, blastocysts, embryo day 6.5, embryo day 7.5, embryo day 9.5, ovary, prostate, vomeronasal organ, tongue, digits, snout epidermis, thymus | Required for import of mRNA binding proteins |
| v-rel reticuloendotheliosis viral oncogene homolog A (avian) (Rela) | NM_009045 | 19 | Adipose tissue, adrenal gland, bone, cerebellum, main olfactory epithelium, fertilized egg, ovary, placenta, prostate, testis, umbilical cord, uterus, oocyte, B220+B-cells, CD4+ Tcells, CD8+ Tcells, lung, lymphnode, vomeronasal organ, tongue, pituitary, digits, epidermis, snout epidermis, spleen, thymus, trachea, bladder, retina | Activation of NF-kappaB transcription factor |
| Pyruvate dehydrogenase kinase, isoenzyme 2 (Pdk2) | NM_133667 | 11 | Brown fat, Adipose tissue, amygdala, frontal cortex, preoptic, cerebellum, cerebral cortex, hippocampus, hypotalamus, main olfactory epithelium, olfactory bulb, spinal cord upper, spinal cord lower, substantia nigra, prostate, testis, heart, large intestine, small intestine, lung, skeletal muscle, vomeronasal organ, tongue, epidermis, snout epidermis, stomach, thymus, thyroid, kidney | Regulation of glucose metabolism |

Table I – *Continued*

| Gene name | GenBank accession | Chromosomes | Predominant expression | Molecular/biological function |
|---|---|---|---|---|
| Toll-like receptor 4 (Tlr4) | NM_021297 | 4 | Brown fat, Adipose tissue, bone, bonemarrow, main olfactory epithelium, mammary gland, prostate, umbilical cord, uterus, heart, large intestine, B220+B-cells, liver, lung, lymphnode, skeletal muscle, vomeronasal organ, salivary gland, tongue, pancreas, digits, epidermis, spleen, stomach, thyroid, trachea, bladder | Activation of NF-kappaB-inducing kinase, catalytic activity, I-kappaB kinase/NF-kappaB cascade, immune response, inflammatory response |
| Zinc finger protein 143 (Zfp143) | NM_009281 | 7 | Brown fat, Adipose tissue, bone, bonemarrow, cerebellum, main olfactory epithelium, embryo day 10.5, embryo day 6.5, embryo day 7.5, embryo day 8.5, embryo day 9.5, fertilized egg, ovary, testis, umbilical cord, uterus, oocyte, heart, B220+B-cells, CD4+ Tcells, CD8+ Tcells, lymphnode, skeletal muscle, vomeronasal organ, salivary gland, digits, snout epidermis, spleen, thymus, thyroid, trachea | Transcriptional activator. binds to the sph motif of small nuclear rna (snRNA) gene promoters |
| Interleukin 2 receptor, gamma chain (Il2rg) | NM_013563 | X | Adipose tissue, bone, bonemarrow, embryo day 6.5, embryo day 7.5, heart, B220+B-cells, CD4+ Tcells, CD8+ Tcells, lung, lymphnode, skeletal muscle, epidermis, spleen, thymus, trachea | Cell surface receptor linked signal transduction |
| UDP-GlcNAc:betaGal beta-1,3-*N*-acetyl-glucosaminyltransferase 1 (B3gnt1) | NM_016888 | 11 | Adipose tissue, adrenal gland, bone, amygdale, frontal cortex, trigeminal, dorsal root ganglia, dorsal striatum, hypotalamus, main olfactory epithelium, embryo day 6.5, fertilized egg, ovary, prostate, uterus, oocyte, large intestine, small intestine, B220+B-cells, CD4+ Tcells, CD8+ Tcells, lung, lymphnode, vomeronasal organ, pituitary, digits, snout epidermis, stomach, thymus, trachea, bladder, kidney | Axon guidance, galactosyltransferase activity, manganese ion binding, protein amino acid glycosylation, sensory perception of smell |
| Splicing factor 3b, subunit 1 (Sf3b1) | NM_031179 | 1 | Adipose tissue, adrenal gland, bonemarrow, amygdale, frontal cortex, preoptic, trigeminal, cerebellum, cerebral cortex, dorsal striatum, hippocampus, hypotalamus, main olfactory epithelium, olfactory bulb, spinal cord upper, spinal cord lower, substantia nigra, embryo day 10.5, embryo day 6.5, embryo day 8.5, embryo day 9.5, fertilized egg, ovary, umbilical cord, uterus, oocyte, heart, B220+B-cells, CD4+ Tcells, lung, pituitary, thymus, trachea, bladder, kidney, retina | Subunit of the splicing factor sf3b required for "a" complex assembly formed by the stable binding of u2 snrnp to the branchpoint sequence (bps) in pre-mRNA |
| Lymphoid enhancer binding factor 1 (Lef1) | NM_010703 | 3 | Brown fat, bonemarrow, blastocysts, embryo day 10.5, embryo day 6.5, embryo day 7.5, embryo day 8.5, embryo day 9.5, fertilized egg, mammary gland, placenta, prostate, testis, umbilical cord, heart, small intestine, B220+B-cells, CD4+ Tcells, CD8+ Tcells, liver, lymphnode, skeletal muscle, salivary gland, pancreas, spleen, stomach, thymus, thyroid, bladder, kidney | Transcriptional activator |

Table I – *Continued*

| Gene name | GenBank accession | Chromosomes | Predominant expression | Molecular/biological function |
|---|---|---|---|---|
| Solute carrier family 12, member 6 (Slc12a6) | NM_133649 | 2E3 | Brown fat, bonemarrow, dorsal striatum, hippocampus, hypotalamus, olfactory bulb, substantia nigra, blastocysts, embryo day 10.5, embryo day 6.5, embryo day 7.5, embryo day 8.5, embryo day 9.5, fertilized egg, mammary gland, placenta, testis, oocyte, heart, large intestine, small intestine, B220+ B-cells, CD4+ Tcells, liver, lymphnode, skeletal muscle, salivary gland, pancreas, epidermis, spleen, stomach, thymus, thyroid, bladder, kidney | Amino acid transport |
| Angio-associated migratory protein (Aamp) | NM_146110 | 1C4 | Adipose tissue, adrenal gland, amygdale, preoptic, cerebellum, cerebral cortex, dorsal root ganglia, hippocampus, hypotalamus, main olfactory epithelium, olfactory bulb, spinal cord lower, substantia nigra, embryo day 6.5, ovary, placenta, prostate, testis, uterus, B220+ B-cells, CD4+ Tcells, CD8+ Tcells, lymphnode, vomeronasal organ, pituitary, digits, snout epidermis, thymus, bladder, kidney | – |
| Nuclear factor of activated T-cells, cytoplasmic, calcineurin-dependent 1 (Nfact1) | NM_016791 | 18 | Brown fat, Adipose tissue, bone, bonemarrow, main olfactory epithelium, fertilized egg, umbilical cord, B220+ B-cells, CD4+ Tcells, CD8+ Tcells, lung, lymphnode, skeletal muscle, vomeronasal organ, salivary gland, tongue, pancreas, digits, epidermis, snout epidermis, spleen, stomach, thymus, thyroid, trachea | Plays a role in the inducible expression of cytokine genes in t cells (by similarity) |
| Actin-binding LIM protein 1 (Ablim1) | NM_178688 | 19 | Brown fat, Adipose tissue, amygdale, preoptic, trigeminal, cerebellum, dorsal root ganglia, olfactory bulb, spinal cord lower, substantia nigra, ovary, umbilical cord, heart, large intestine, B220+ B-cells, CD4+ Tcells, CD8+ Tcells, lung, lymphnode, vomeronasal organ, tongue, digits, epidermis, snout epidermis, spleen, stomach, thymus, thyroid, trachea, retina | Actin binding, axon guidance, cytoskeleton organization and biogenesis, metal ion binding, zinc ion binding |
| DNA methyltransferase 3A (Dnmt3a) | NM_153743 | 12 A2–A3 | Adipose tissue, bonemarrow, amygdale, preoptic, cerebellum, cerebral cortex, dorsal striatum, hippocampus, hypotalamus, main olfactory epithelium, olfactory bulb, spinal cord upper, spinal cord lower, substantia nigra, embryo day 10.5, ovary, placenta, umbilical cord, uterus, heart, lymphnode, skeletal muscle, vomeronasal organ, pancreas, pituitary, snout epidermis, spleen, thymus, thyroid, retina | Required for genome wide de novo methylation and is essential for development |

Table I – *Continued*

| Gene name | GenBank accession | Chromosomes | Predominant expression | Molecular/biological function |
|---|---|---|---|---|
| Early growth response 1 (Egr1) | NM_007913 | 18 | Adipose tissue, adrenal gland, amygdale, frontal cortex, preoptic, cerebellum, cerebral cortex, dorsal root ganglia, hippocampus, hypotalamus, olfactory bulb, spinal cord lower, substancia nigra, ovary, heart, small intestine, B220+B-cells, CD4+ Tcells, CD8+ Tcells, lung, lymphnode, skeletal muscle, tongue, pituitary, digits, epidermis, snout epidermis, stomach, thymus, trachea | Transcriptional regulator |
| Deoxyhypusine synthase (Dhps) | NM_201408 | 8C2 | Bonemarrow, amygdale, frontal cortex, preoptic, trigem-inal, cerebellum, cerebral cortex, dorsal root ganglia, dorsal striatum, hippocampus, hypotalamus, olfactory bulb, substantia nigra, blastocysts, embryo day 10.5, embryo day 6.5, embryo day 7.5, embryo day 8.5, embryo day 9.5, mammary gland, uterus, B220+B-cells, CD4+ Tcells, CD8+ Tcells, lymphnode, vomeronasal organ, thymus, kidney, retina | Catalyzes the NAD-dependent oxidative cleavage of spermidine |
| Insulin degrading enzyme (Ide) | NM_031156 | 19 | Adipose tissue, adrenal gland, main olfactory, embryo day 6.5, embryo day 7.5, embryo day 9.5, fertilized egg, ovary, prostate, uterus, oocyte, heart, CD8+ Tcells, skeletal muscle, vomeronasal organ, salivary gland, tongue, thymus, trachea, bladder | Can cleave insulin and tgf-alpha |
| Interleukin 12a (IL12a) | NM_008351 | 3 | Brown fat, bone, bonemarrow, preoptic, cerebellum, cerebral cortex, dorsal striatum, olfactory bulb, spinal cord upper, substantia nigra, embryo day 10.5, embryo day 6.5, embryo day 7.5, embryo day 8.5, embryo day 9.5, fertilized egg, B220+B-cells, CD8+ Tcells, lymphnode, skeletal muscle, spleen, thyroid | Cytokine that can act as a growth factor for activated T and NK cells (by similarity) |
| Helicase, mus308-like (Drosophila) (Hel308) | BC082601 | 5E | Fertilized egg, oocyte | ATP binding, DNA metabolism, helicase activity, hydro-lase activity, RNA binding, single-stranded DNA-dependent ATP-dependent DNA helicase activity |
| Nuclear receptor subfamily 3, group C, member 1 (Nr3c1) | NM_008173 | 18 | Brown fat, Adipose tissue, frontal cortex, preoptic, cerebellum, main olfactory epithelium, ovary, placenta, umbilical cord, large intestine, B220+B-cells, CD4+ Tcells, CD8+ Tcells, lung, skeletal muscle, epidermis, thymus, trachea, kidney | Receptor for glucocorticoids (gc) |

### Determination of PGE in the thymus

Promiscuous gene expression is currently identified on the basis of data from microarray analysis for the different mouse organs using combined information from the public database GNF Gene Expression Atlas (http://symatlas.gnf.org/SymAtlas) (Su et al. 2004). This data bank presents gene expression in more than 60 mouse tissues/organs as assessed by gene array analysis using Affymetrics microarrays. Data information include GenBank accession, chromosomal location and molecular/biological function of each gene analysed (Table I).

At present, we are considering only the promiscuous genes whose expression was significantly induced in the thymus (as displayed by the SAM algorithm) and detected in different organs or tissues, besides the thymus, and whose expression levels were greater than median in relation to all other organs which appear in the GNF Atlas.

### Emergence of PGE during thymus ontogeny

The complexity of PGE depends on increases in ascending order from cTECs, to immature mTECs, to mature $CD80^{hi}$ mTECs. These different gene pools are not complementary but additive, that is, there is no apparent association between the respective molecular/biological functions of the genes in parenchymal organs. The significance of PGE in the thymus is associated with central tolerance (Sospedra et al. 1998; Bruno et al. 2002, 2004) persisting during the entire exporting period of T cells from the thymus (Derbinski et al. 2001).

In fact, self-tolerance induction is dependent on developmentally regulated key processes, such as expression of TCR on the surface of lymphocytes allowing for TCR–MHC–peptide recognition, which enables the positive/negative selection of T cells in fetal thymus (Kyewski and Derbinski 2004).

Studies with freshly obtained fetal thymus allowed for the demarcation of the emergence of TCR beta V(D)J recombination during *in vivo* thymus ontogeny among different inbred mouse strains, contributing to revealing the effect of the genetic background on T cell development (Macedo et al. 1999; Espanhol et al. 2004; Cardoso et al. 2006).

The control of gene expression during the development of this organ has gained priority among several research teams, including our own group (Espanhol et al. 2004; Magalhães et al. 2005; Cardoso et al. 2006) with the objective of developing a clearer understanding the molecular mechanism of the central tolerance induction.

The cDNA microarray method has permitted the possibility of analysing thousands of genes at once and of performing a true dissection of this organ by means of transcript identification, characterizing virtually all the main cell types populating the thymus (Puthier et al. 2004).

In a recent study, using the cDNA microarray method, we identified the *in vivo* modulation of several cell-signaling genes, including those of the calcium cascade pathway, which is important for individual stages of T cell maturation and the control of anergy during murine thymus ontogeny (Puthier et al. 2004).

Using the same kind of analysis, our group recently showed the modulation of gene expression in murine fetal thymus organ cultures (FTOCs), at transcriptome scale and revealed an overlap between genes associated with TCR V(D)J recombination and DNA repair. We demonstrated that the association of FTOC cultures and cDNA microarray technology allows for sufficient accuracy to uncover the participation of essential genes implicated in thymus development (Cardoso et al. 2006)

Considering that the expression of TCR in the surface of maturing T cells is a key feature, allowing the recognition of MHC-peptide during positive/negative selection, we are currently employing cDNA microarrays to observe the extent of PGE when the TCR V beta gene (TRBV8.1) rearrangement emerges during the *in vivo* development of the thymus of Balb-c, C57Bl/6 and (Balb-c × c57Bl/6)$F_1$ mouse strains.

Comparing thymus gestation time as a result of fetal age, it was possible to determine the onset of gene induction, representing 57 different parenchymal and 7 lymphoid organs whose coded proteins are considered to be parenchymal organ antigens, thus indicating the occurrence of PGE.

Thymus transcriptome profiling was assessed using glass slide cDNA microarrays containing 4500 IMAGE thymus target sequences hybridized with fluorescent Cy3 or Cy5 cDNA probes. PGE was identified on the basis of gene expression data for the different parenchymal organs.

To identify significant changes in the gene expression of fetal thymus when TRBV8.1 V(D)J recombination occurred compared to a reference pool, we used a scatter plot of the observed relative difference $d(i)$ vs. the expected relative difference $d_E(i)$ as shown by the SAM program.

Most of the 4500 sequences tested presented $d(i) \cong d_E(i)$, indicating that their expression pattern remained unaltered. However, some genes were significantly repressed (71 in Balb-c, 57 in C57Bl/6 and 17 genes in (Balb-c × C57Bl/6)$F_1$) or induced (3 in C57Bl/6 and 70 genes in (Balb-c × C57Bl/6)$F_1$).

In this study, we defined only the induced genes as a manifestation of PGE (Table I). Moreover, 2 induced ESTs were found in C57Bl/6 and 38 in (Balb-c × C57Bl/6)$F_1$ but, due to their actual status of unknown function or organ representation, these sequences were not considered. This is evidence that the extent of PGE is greater than observed in this study.

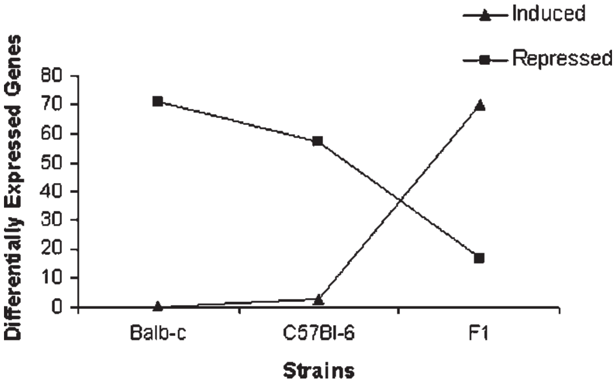

Figure 1. Number of significant induced or repressed genes in the fetal thymus during the emergence of TRBV8.1 recombination among inbred mouse strains. F1 = (Balb-c × C57Bl/6)$F_1$.

Interestingly, the microarray design and statistical stringency used in the SAM program allowed us to observe the manifestation of PGE at the onset of TRVB8.1 V(D)J recombination but this was observed only in the C57Bl/6 strain and the (Balb-c × C57Bl/6)$F_1$ hybrid. In the same developmental period, the Balb-c strain did not exhibit PGE, considering the statistical stringency we used in this study (Table I).

Figure 1 shows that among the strains studied, significant induction is inversely proportional to gene repression, strongly suggesting a role for the genetic background of strains in the control of PGE. Figure 2 illustrates that the reproductive and central nervous systems and stem cells/glands in C57Bl/6 and the central nervous and reproductive systems and stem cells in (Balb-c × C57Bl/6)$F_1$ are the predominant parenchymal organs most represented in the thymus in this phase of development, followed by the lymphoid system.

To our knowledge, these findings represent the first association study between the emergence of a TR gene V(D)J recombination (TRVB8.1) and occurrence of PGE among inbred mouse strains, with implications regarding the fine demarcation of key processes of self-tolerance induction.

### Parenchymal organ representation in the thymus

The 3 induced genes in C57Bl/6 and 71 in (Balb-c × C57Bl/6)$F_1$ identified as significantly induced at 14–15 days p.c. were assigned to 57 parenchymal and 7 lymphoid organs according to their predominant expression, which were subgrouped in 17 systems.

### Chromosomal location of the differentially expressed genes

The genomic distribution of the significantly modulated genes (repressed and induced), 71 in Balb-c, 60 in C57Bl/6 and 87 in (Balb-c × C57Bl/6)$F_1$, allowed for the organization of chromosomal clusters of coordinated expression.

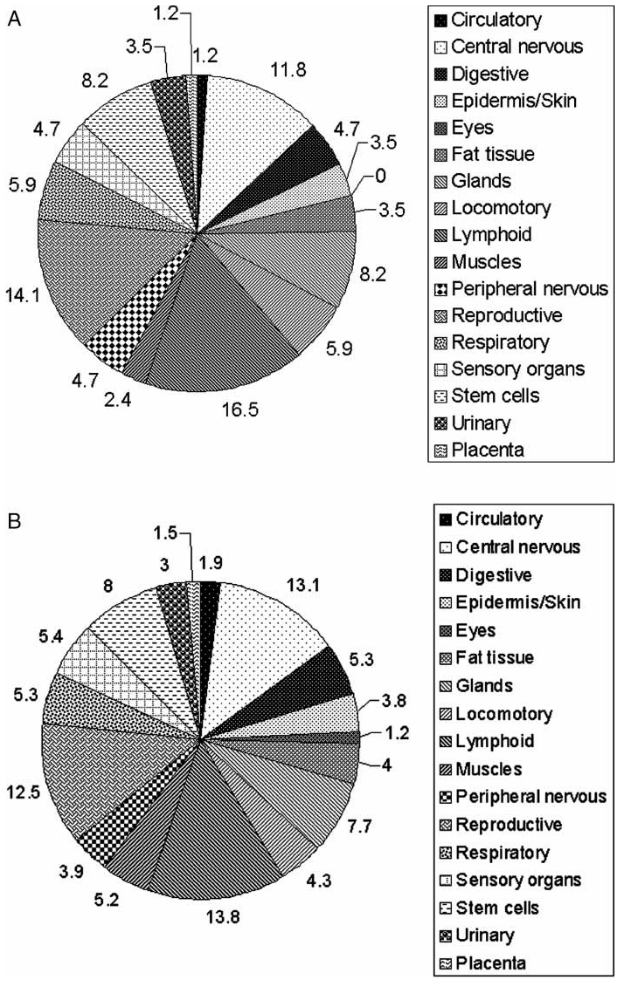

Figure 2. Representation of tissue/organ systems specific gene expression in the fetal thymus during the emergence of TRBV8.1 recombination. Analysis of 4500 sequences was performed using glass slide cDNA microarrays, whose significant induced genes were annotated characterizing the promiscuous expression, which allow self-representation of tissue specific antigens in the thymus. 2A = C57Bl/6, 2B = (Balb-c × C57Bl/6)$F_1$.

Figure 3 shows the frequency distribution of the repressed and induced genes among chromosomes. All chromosomes, except Y, harbor differentially expressed genes, with slightly biased distribution on chromosomes 2, 5, 11, 13, 17 and 19 for the repressed genes in Balb-c, on chromosomes 2, 3, 6, 9 and 11 for the repressed and 11, 13 and 17 for the induced in C57Bl/6, on chromosomes 2, 7, 9, 13 and 15 for the repressed and on chromosomes 2, 4, 5, 7, 10, 11, 18 and 19 for the induced genes in the (Balb-c × C57Bl/6)$F_1$ hybrid.

### Discussion and perspective

Self-tolerance induction should occur early, during the fetal development of the thymus, preventing

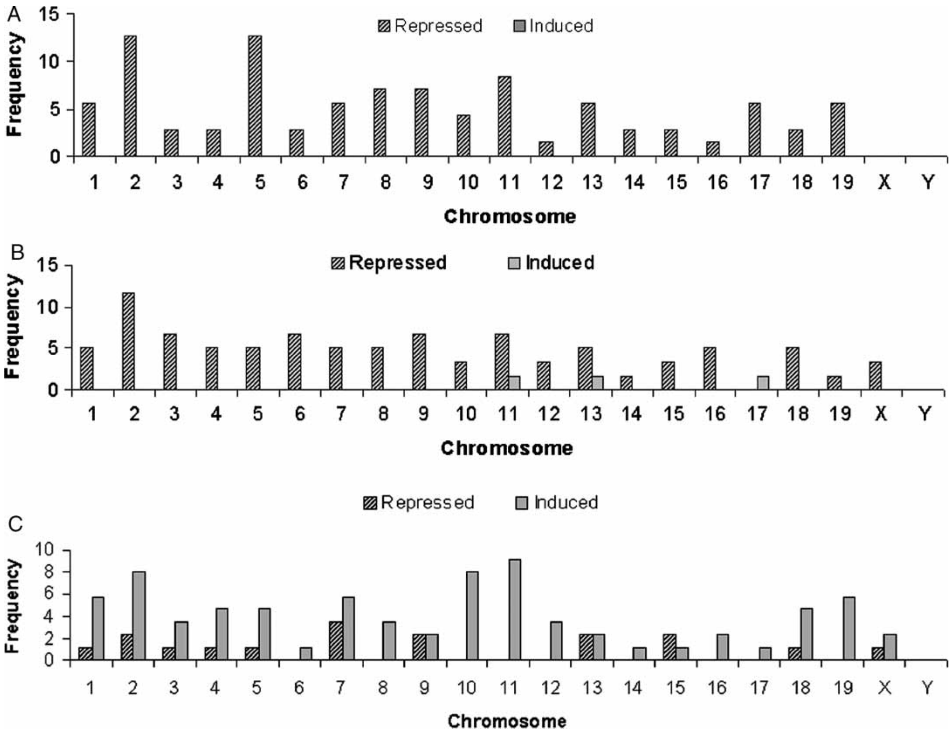

Figure 3. Chromosomal distribution of the repressed and induced genes in the fetal thymus during the emergence of TRBV8.1 recombination among inbred mouse strains. 3A = Balb-c, 3B = C57Bl/6, 3C = (Balb-c × C57Bl/6)$F_1$.

autoimmune pathological reactions. This phenomenon is dependent on the presentation of self-antigens to maturing T cells through TCR-MHC-peptide recognition (Sprent and Webb 1995; Hanahan 1998; Kishimoto and Sprent 2000). The assembling of functional TR genes, allowing for the expression of TCR on the surface of T cells, is dependent on the temporal emergence of TR gene V(D)J recombination, during the fetal development of the thymus (Junta and Passos 1998; Macedo et al. 1999; Espanhol et al. 2004).

The extent of self-representation of most parenchymal tissues and organs is guaranteed by PGE in the thymus, a phenomenon that is exhibited by mTECs, is complex and involves 5–10% of all known genes in mice and humans. Accordingly, the complexity of PGE increases in ascending order, from cTECs to immature mTECs to mature $CD80^{hi}$ mTECs. These different gene pools are not complementary but additive, that is, there is no apparent association between the respective molecular/biological function of the genes in parenchymal organs. The significance of PGE in the thymus is associated with central tolerance of T cells (Sospedra et al. 1998; Bruno et al. 2002; Kyewski and Derbinski 2004). While PGE by mTECs was well characterized by the authors cited above, its time course during thymus ontogenetic development requires further exploration.

Molecular characterization of PGE during fetal development of different inbred mouse strains is a relevant approach in immunobiology, since this system can represent a potential tool in clarifying this question and in evaluating whether the different genetic backgrounds play a role in the control of PGE.

Moreover, knockout (KO) mice could reveal evidence for the role of specific genes in this process.

Evidence obtained by our team shows, for the first time, the occurrence of PGE *in vivo* during the fetal development of the thymus. To evaluate whether PGE in this model system is a developmental dependent phenomenon, we regarded the differential gene expression during ontogeny through the comparison of gestation days (p.c.), as the most informative in the delineation of the gene pool.

Moreover, we compared two different inbred mouse strains expressing different MHC haplotypes, Balb-c = H-2d and C57Bl/6 = H-2K and their hybrid (Balb-c × C57Bl/6)$F_1$ demonstrating that PGE emerges on different days among the strains studied. These findings strongly suggest a role for the genetic background of these strains in the control of the emergence and extent of PGE.

Our results show that PGE occurs during thymus maturation; after TRBV8.1 gene V(D)J recombination in Balb-c and coinciding with TRBV8.1 V(D)J recombination in C57Bl/6 and in (Balb-c × C57Bl/6)$F_1$.

The early fetal thymus, by 13–15 days p.c., is mainly composed of homogeneous double-negative (DN) $CD4^-CD8^-$T cell precursors. By day 18 p.c. this population gradually acquires the CD4 marker resembling the adult CD4$^{low}$ precursor (Shortman and Wu 1996).

These features allow for TCR-MHC-peptide recognition and enable the positive/negative selection of T cells in fetal thymus.

Our evidence for the occurrence of PGE in the late fetal thymus can be associated with the timing of the molecular events of T cell tolerance induction during ontogeny.

The data collected here were obtained by the cDNA microarray method, with the expression of the 4500 mouse mRNA sequences analyzed by the SAM algorithm (20). We found statistically significant gene modulation showing 71 repressed genes in Balb-c, 57 repressed and 3 induced genes in C57Bl/6 and 17 repressed and 70 induced genes in (Balb-c × C57Bl/6)$F_1$. Moreover, the significantly induced genes were indicative of emergence of PGE (Table I).

While the experiments were not conducted with cell-sorted purified mTECs, this caused no problems in the detection of differential TSA gene expression in the thymus. In order to bypass this potential difficulty, we used a cDNA microarray method, including a dedicated statistical algorithm for data analysis (SAM algorithm), which presented sufficient accuracy to distinguish and quantify TSA gene expression originating from thymic epithelial cells, especially mTECs (Gotter et al. 2004, Kyewski and Derbinski 2004; Derbinski et al. 2005).

In fact, cDNA microarray data mining has permitted the virtual dissection of the mouse thymus into its principal cellular components by means of the identification of the specific cellular transcripts (mRNAs) (Puthier et al. 2004). These observations demonstrate the feasibility of the use of whole thymus as starting material in PGE studies.

In agreement with previous observations (Derbinski et al. 1995) the molecular/biological function of promiscuously expressed genes found in our model system, showed no interrelationship (Table I).

Regarding the chromosomal localization of the repressed and induced genes, no important preferential distribution was identified. All chromosomes harbor promiscuously expressed genes with slightly biased distribution on chromosomes 2, 5, 10 and 11 among the repressed genes and on chromosomes 2, 10 and 11 among the induced genes. The exception was chromosome Y on which no repressed or induced gene was positioned considering the statistical stringency used in this study. (Figure 3).

This feature of random PGE distribution in the genome, strongly suggests an uncommon model of gene regulation found in the thymus that needs further study.

In this regard, certain urgent questions remain, like whether the chromatin scaffold in mTECs is so arranged to allow for promiscuous gene expression and whether the methylation pattern of mouse genome controls this phenomenon.

Finally, the use of the fetal thymus model system and cDNA microarray method, open up perspectives to determine the modulation and extent of PGE in autoimmune diseases, through the analysis of genetically compromised mouse strains.

During preparing this article, the Science magazine (Sciencexpress Report) published online the evidence for a functional second thymus in mice, located in the neck, whose observations were done and communicated by Dr Rodewald's group of the University of Ulm, Ulm, Germany (Terszowski et al. 2006). Once broadly recognized the existence of the second thymus, this observation also provide perspective for reevaluating the physiological relevance of extra-thymic T cell development, as stated the authors and evaluate the occurrence of PGE in this organ.

### Acknowledgements

Our research group received financial support from the FAPESP (Fundação de Amparo à Pesquisa do Estado de São Paulo, Brasil) and the CNPq (Consellho Nacional de Desenvolvimento Científico e Tecnológico, Brasil). The 2NTB cDNA library used to prepare the microarrays was kindly ceded by Dr Catherine Nguyen of the INSERM (Institut National de la Santé et de la Recherche Médicale, ERM 206, Marseille, France).

### References

Alferink J, Aigner S, Reibke R, Hämmerling GJ, Arnold B. 1999. Peripheral T cell tolerance: The contribution of permissive T cell migration into parenchymal tissues of the neonate. Immunol Rev 169:255–261.

Bruno R, Sabater L, Sospedra M, Ferrer-Francesch X, Escudero D, Martínez-Cáceres E, Pujol-Borrel R. 2002. Multiple sclerosis candidate autoantigens except myelin oligodendrocyte glycoprotein are transcribed in the human thymus. Eur J Immunol 32:2737–2747.

Bruno R, Sabater L, Tolosa E, Sospedra M, Ferrer-Francesch X, Coll J, Foz M, Melms A, Pujol-Borrell R. 2004. Different patterns of nicotinic acetylcholine receptor subunit transcription in human thymus. J Neuroimmunol 149:147–159.

Cardoso RS, Junta CM, Macedo C, Magalhães DAR, Silveira ELV, Paula MO, Marques MMC, Mello SS, Zarate-Blades CR, Nguyen C, Houlgatte R, Donadi EA, Sakamoto-Hojo ET, Passos GAS. 2006. Hybridization signatures of gamma-irradiated murine fetal thymus organ culture (FTOC) reveal modulation of genes associated with T-cell receptor V(D)J recombination and DNA repair. Mol Immunol 43:464–472.

Derbinski J, Schulte A, Kyewski B, Klein L. 2001. Promiscuous gene expression in medullary thymic epithelial cells mirrors the peripheral self. Nat Immunol 2:1032–1039.

Derbinski J, Gäbler J, Brors B, Tierling S, Jonnakuty S, Hergenhahn M, Peltonen L, Walter J, Kyewski B. 2005. Promiscuous gene expression in thymic epithelial cells is regulated at multiple levels. J Exp Med 202:33–45.

Ehrlich P, Morgenroth J. 1901. Ueber Hämolysine. Berl Klin Wochenschr 28:251–257.

Espanhol AR, Cardoso RS, Junta CM, Victorero G, Loriod B, Nguyen C, Passos GAS. 2004. Large scale gene expression analysis of CBA/J mouse strain fetal thymus using cDNA-array hybridizations. Mol Cell Biochem 206:65–68.

Gallegos A, Bevan MJ. 2006. Central tolerance: Good but imperfect. Immunol Rev 209:290–296.

Gotter J, Brors B, Hergenhahn M, Kyewski B. 2004. Medullary epithelial cells of the human thymus express a highly diverse selection of tissue-specific genes co-localized in chromosomal clusters. J Exp Med 199:155–166.

Hanahan D. 1998. Peripheral-antigen-expressing cells in thymic medulla: Factors in self-tolerance and autoimmunity. Curr Opin Immunol 10:656–662.

Jolicoeur C, Hanahan D, Smith KM. 1994. T-cell tolerance toward a transgenic beta-cell antigen and transcription of endogenous pancreatic genes in thymus. Proc Natl Acad Sci USA 91:6707–6711.

Junta CM, Passos GAS. 1998. Emergence of TCRαβ V(D)J recombination and transcription during thymus ontogeny of inbred mouse strains. Mol Cell Biochem 187:67–72.

Kishimoto H, Sprent J. 2000. The thymus and negative selection. Immunol Res 21:315–323.

Klein L, Kyewski B. 2000. Self-antigen presentation by thymic stromal cells: A subtle division of labor. Curr Opin Immunol 12:179–186.

Kyewski B, Derbinski J. 2004. Self-representation in the thymus: An extended view. Nat Rev Immunol 4:688–698.

Kyewski B, Derbinski J, Gotter J, Klein L. 2002. Promiscuous gene expression and central T-cell tolerance: More than meets the eye. Trends Immunol 23:364–371.

Macedo C, Junta CM, Passos GAS. 1999. Onset of T-cell receptor Vβ8.1 and Dβ2.1 V(D)J recombination during thymus development of inbred mouse strains. Immunol Lett 69:371–373.

Magalhães DA, Macedo C, Junta CM, Mello SS, Marques MM, Cardoso RS, Sakamoto-Hojo ET, Passos GAS. 2005. Hybridization signatures during thymus ontogeny reveals modulation of genes coding for T-cell signaling proteins. Mol Immunol 42:1043–1048.

Medzhitov R, Janeway CA. 1997. Innate immunity, the virtues of a monoclonal system recognition. Cell 91:295–298.

Nguyen C, Rocha D, Granjeaud S, Baldit M, Bernard K, Naquet P, Jordan BR. 1995. Differential gene expression in the murine thymus assayed by quantitative hybridization of arrayed cDNA clones. Genomics 29:207–215.

Puthier D, Joly F, Irla M, Saade M, Victorero G, Loriod B, Nguyen C. 2004. A general survey of thymocyte differentiation by transcriptional analysis of knockout mouse models. J Immunol :6109–6118.

Rugh R. 1968. The mouse. Its reproduction and development. 1st ed. Edina, MN, USA: Burgess Publishing Company.

Sakamoto-Hojo ET, Passos GAS. 2006. Hybridization signatures of gamma-irradiated murine fetal thymus organ culture (FTOC) reveal modulation of genes associated with T-cell receptor V(D)J recombination and DNA repair. Mol Immunol 43:464–472.

Schwartz RH, Mueller DL. 2003. In: Paul WE, editor. Fundamental immunology. 5th ed., Philadelphia: Lippincott Williams & Wilkins. p 901–934.

Shortman K, Wu L. 1996. Early T lymphocyte progenitors. Annu Rev Immunol 14:29–47.

Sospedra M, Ferrer-Francesch X, Dominguez O, Juan M, Foz-Sala M, Pujol-Borrel R. 1998. Transcription of a broad range of self-antigens in human thymus suggests a role for central mechanisms in tolerance toward peripheral antigens. J Immunol 161:5918–5929.

Sprent J, Webb SR. 1995. Intrathymic and extrathymic clonal deletion of T-cells. Curr Opin Immunol 7:196–205.

Su AI, Wiltshire T, Batalov S, Lapp H, Ching KA, Block D, Zhang J, Soden R, Hayakawa M, Kreiman G, Cooke MP, Walker JR, Hogenesch JB. 2004. A gene atlas of the mouse and human protein-encoding transcriptomes. Proc Natl Acad Sci USA 101:6062–6067.

Terszowski G, Müller S, Bleul CC, Blum C, Schirmbeck R, Reimamm J, DuPasquier L, Amagai T, Boehm T, Rodewald H-R. 2006. Evidence for a second thymus in mice. Science, (Sciencexpress, www.sciencexpress.org, 2 March 2006; 10.1126/science.1123497).

Tusher VG, Tibshirani R, Chu G. 2001. Significance analysis of microarrays applied to the ionizing radiation response. Proc Natl Acad Sci USA 98:5116–5121.

Walker LS, Abbas AK. 2002. The enemy within: Keeping self-reactive T cells at bay in the periphery. Nat Rev Immunol 2:11–19.

